# Tissue damage by laser radiation: an in vitro comparison between Tm:YAG and Ho:YAG laser on a porcine kidney model

**DOI:** 10.1186/s40064-016-1750-3

**Published:** 2016-03-03

**Authors:** Stephan Huusmann, Mathias Wolters, Mario W. Kramer, Thorsten Bach, Heinrich-Otto Teichmann, Andreas Eing, Sebastian Bardosi, Thomas R. W. Herrmann

**Affiliations:** Department of Urology and Urological Oncology, Hannover Medical School (MHH), Carl Neuberg Str. 1, 30625 Hannover, Germany; Department of Urology, Asklepios Hospital Harburg, Eissendorfer Pferdeweg 52, 21075 Hamburg, Germany; LISA Laser Products OHG, Max-Planck-Strasse 1, 37191 Katlenburg-Lindau, Germany; MVZ wagnerstibbe Pathologie, Neuropathologie und Laboratoriumsmedizin, An der Lutter 24, 37075 Göttingen, Germany; Department of Urology, Clinic of the University of Schleswig Holstein / Campus Luebeck, Ratzeburger Allee 160, 23538 Lübeck, Germany

**Keywords:** Tm:YAG, Ho:YAG, Laser effects, Tissue damage

## Abstract

The understanding of tissue damage by laser radiation is very important for the safety in the application of surgical lasers. The objective of this study is to evaluate cutting, vaporization and coagulation properties of the 2 µm Tm:YAG laser (LISA Laser Products OHG, GER) in comparison to the 2.1 µm Ho:YAG laser (Coherent Medical Group, USA) at different laser power settings in an in vitro model of freshly harvested porcine kidneys. Laser radiation of both laser generators was delivered by using a laser fiber with an optical core diameter of 550 µm (RigiFib, LISA Laser GER). Freshly harvested porcine kidneys were used as tissue model. Experiments were either performed in ambient air or in aqueous saline. The Tm:YAG laser was adjusted to 5 W for low and 120 W for the high power setting. The Ho:YAG laser was adjusted to 0.5 J and 10 Hz (5 W average power) for low power setting and to 2.0 J and 40 Hz (80 W average power) for high power setting, accordingly. The specimens of the cutting experiments were fixed in 4 % formalin, embedded in paraffin and stained with Toluidin blue. The laser damage zone was measured under microscope as the main evaluation criteria. Laser damage zone consists of an outer coagulation zone plus a further necrotic zone. In the ambient air experiments the laser damage zone for the low power setting was 745 ± 119 µm for the Tm:YAG and 614 ± 187 µm for the Ho:YAG laser. On the high power setting, the damage zone was 760 ± 167 µm for Tm:YAG and 715 ± 142 µm for Ho:YAG. The incision depth in ambient air on the low power setting was 346 ± 199 µm for Tm:YAG, 118 ± 119 µm for Ho:YAG. On the high power setting incision depth was 5083 ± 144 µm (Tm:YAG) and 1126 ± 383 µm (Ho:YAG) respectively. In the saline solution experiments, the laser damage zone was 550 ± 137 µm (Tm:YAG) versus 447 ± 65 µm (Ho:YAG), on the low power setting and 653 ± 137 µm (Tm:YAG) versus 677 ± 134 µm (Ho:YAG) on the high power setting. Incision depth was 1214 ± 888 µm for Ho:YAG whereas Tm:YAG did not cut tissue at 5 W in saline solution. On the high power setting, the incision depth was 4050 ± 1058 µm for Tm:YAG and 4083 ± 520 µm for Ho:YAG. Both lasers create similar laser damage zones of <1 mm in ambient air and in saline solution. These in vitro experiments correspond well with in vivo experiments. Thereby, Tm:YAG offers a cutting performance, coagulation and safety profile similar to the standard Ho:YAG lasers in urological surgery.

## Background

Over the past few years, several laser devices were introduced in urologic surgery. The pulsed holmium:YAG (Ho:YAG) laser, with a wavelength of 2123 nm, is the most commonly used laser because of its wide field of application regarding tissue surgery and stone treatment. This energy source has become the standard tool for intracorporal lithotripsy in percutaneous or retrograde intrarenal surgery (Gupta 2007; Pierre and Preminger 2007). With water as the chromophore the Ho:YAG laser radiation is highly absorbed in any water containing material, such as tissue or urinary stones, leading to an instant vaporization and a low penetration depth (Herrmann et al. 2012; Teichmann et al. 2007).

The continuous wave thulium:YAG (Tm:YAG) laser, with a wavelength of 2013 nm, was introduced as an alternative laser for tissue surgery, especially for prostate surgery (Bach et al. 2007, 2010; Fried and Murray 2005). In addition, the Tm:YAG laser radiation is absorbed by water molecules. Further ex vivo studies proved increased tissue ablation capacity, shallower and even tissue penetration depth, and equal hemostatic properties when compared to the potassium-titanyl-phosphate-laser (KTP) and lithium-borate-laser (LBO) (Herrmann et al. 2012; Bach et al. 2010; Heinrich et al. 2010; Wendt-Nordahl et al. 2008).

In the present in vitro study, the Tm:YAG laser (RevoLix 120 Thulium laser, LISA Laser Products OHG, Katlenburg-Lindau, Germany) was compared, with regard to the cutting, vaporization, and coagulation properties, to the Ho:YAG laser (VersaPulse Select Holmium laser, Coherent Medical Group, USA) as reference standard at different laser power settings on freshly harvested porcine cadaver kidneys. Based on the different physical properties of each laser system, additional ex vivo experiments are necessary to understand the effect on tissue and to assess the complications and the clinical outcome after surgery. The aim was to clarify whether the Tm:YAG laser offers comparable characteristics which may lead to comparable safety for surgical application. The data of this study was used to formally clear the acceptance of the medical board of japan guaranteeing efficacy and safety for the Tm:YAG laser with regard to the commonly accepted Ho:YAG laser.

## Methods

The Tm:YAG laser (RevoLix 120 Thulium laser, LISA Laser Products OHG, Katlenburg-Lindau, Germany) emits laser radiation at a wavelength of 2013 nm in continuous wave fashion. The laser power is directly set in watt (W) because of the continuous wave mode.

The Ho:YAG laser (VersaPulse Select Holmium laser, by Coherent Medical Group, USA) emits laser radiation at a wavelength of 2123 nm in a pulsed manner. The output of the Ho:YAG laser is set separately for pulse energy in joule (J) and pulse repetition rate in Hertz (Hz). The multiplication of pulse energy (J) and repetition rate (Hz) is the average output power in watt (W).

For delivery of laser radiation a laser fiber with an optical core diameter of 550 µm (RigiFib laser fiber, LISA, Katlenburg-Lindau, Germany) was used for both laser units. The fiber was cut prior to every single experiment by a scissor.

For handling purposes a handpiece guiding the laser fiber (SurgiLas 110-1.1, LISA Laser Products OHG, Katlenburg-Linda, Germany) was used.

Kidneys were harvested from freshly slaughtered pigs, rinsed in water and stored at 4–8 °C until the experiments were performed.

All results are given as arithmetic mean of three equal experiments for every setting.

### Experiments in ambient air

Laser delivery to the tissue was carried out in a standardized setup. The tissue was cut under visual guidance. The angle of the handpiece with the fiber to the kidney surface was exactly 45°. Cutting was performed with a defined translation speed of 2 mm/s. The distance between the fiber tip and the tissue was 0.5 mm. Three incisions with a length of 1 cm were performed on each power setting and classified for later identification.

For the histological preparation, the tissue samples were cut with a cold knife perpendicular to the laser incision and fixed in vials of 4 % formalin for 48 h.

The laser settings for the cutting experiments consisted of a low power and a high power setting. For the low power setting, the Tm:YAG laser was set to a laser power of 5 W. The Ho:YAG laser was set to 0.5 J pulse energy with a repetition rate of 10 Hz which multiplies to an average laser power of 5 W. On the high power setting, the Tm:YAG laser was set to the maximum power of 120 W of the Revolix generator. The Ho:YAG laser was set to 2.0 J pulse energy with a 40 Hz repetition rate multiplying to 80 W average laser power which represents the maximum of the VersaPulse Select generator.

### Experiments in aqueous saline solution

For the experiments in aqueous saline solution, a transparent basin was filled with 0.9 % NaCl aqueous solution at 23 °C. The kidneys were fixed to the bottom of the basin. The laser incisions were conducted in the same way as described in the laser incision experiments in ambient air. The power settings for the aqueous saline solution experiments were identical to those as used in the experiments in ambient air including a low power and a high power setting.

#### Histological evaluation of tissue effect

The depth of tissue vaporization (laser incision) and the width of the damage zones were measured under a microscope using a calibrated caliper.

For the histological evaluation the specimens was fixed in 4 % formalin, they were embedded in paraffin. Serial slices with a thickness of 2–3 µm were prepared and stained with toluidine blue, which had an advantage in circumscribing the E-zone compared to haematoxylin and eosin stain.

### Items: OC-zone, NT-zone, E-zone

**Fig. 1:**
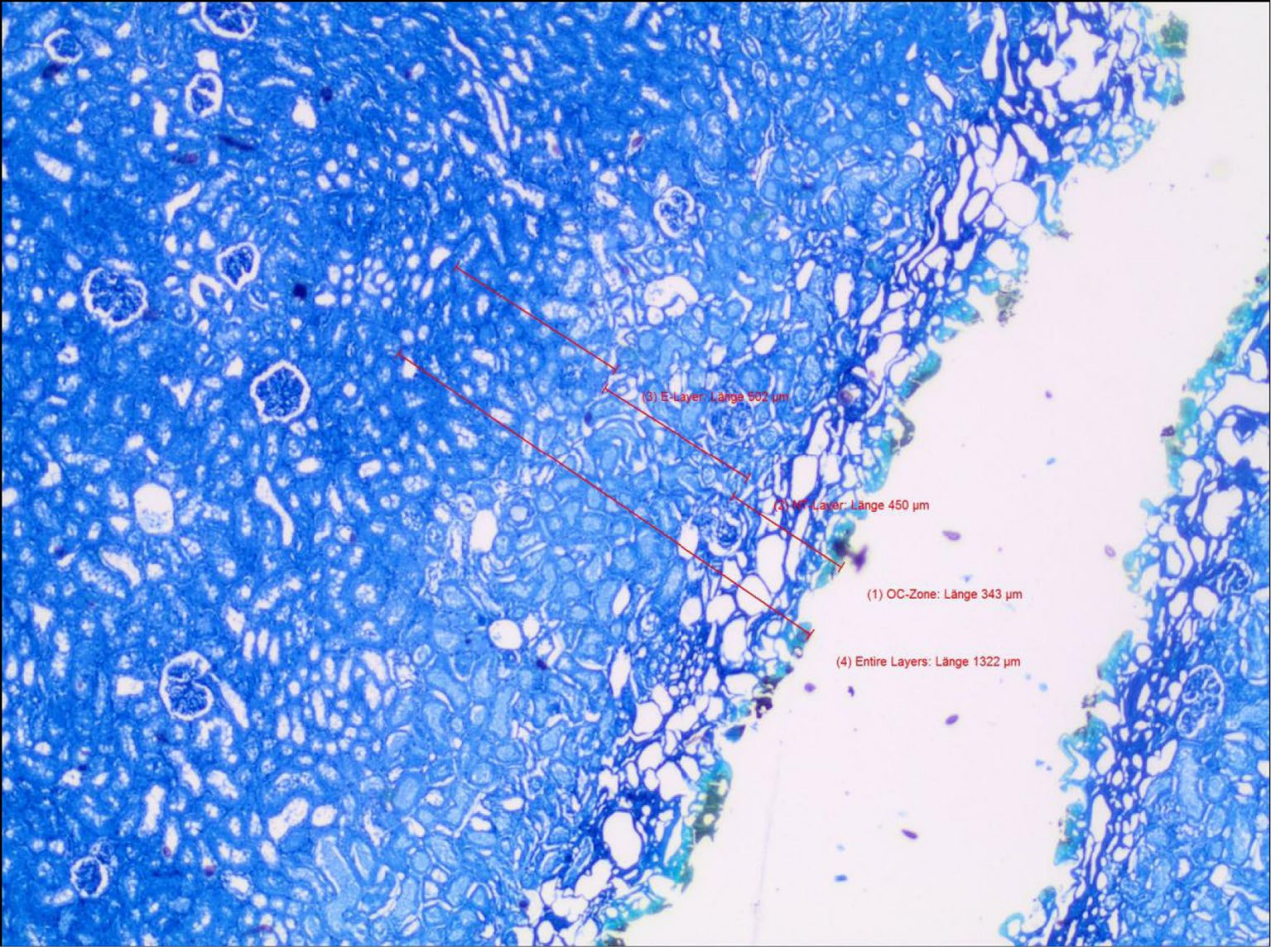
Histologic specimen of a laser cut in porcine kidney by Tm:YAG at 120 W power in ambient air

The outer coagulation zone (OC-zone) is defined as a carbonized seam and a tissue layer with vacuolization underneath. In toluidine blue staining, the OC-zone appears green blue or dark blue (Fig. 1). The necrotic tissue layer (NT-zone) is light blue in images. At high magnification, the pycnotic nuclei of the cells may be observed. The subsequent edema zone (E-zone) results from exposure to heat generated from absorbed laser energy. In vivo, the E-zone has the potential to recover thus it is considered not to be part of the laser damage zone. In histologic images it is slightly darker blue than the healthy tissue (Fig. 1).

**Fig. 2:**
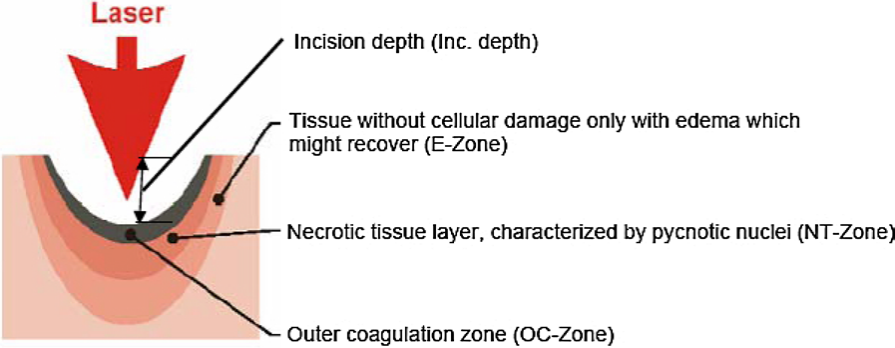
Laser affected tissue layers after laser irradiation

By definition the laser damage zone consists of the outer coagulation zone plus the necrotic zone (Fig. 2).

Furthermore the incision depth created by the vaporization of tissue due to absorption of laser radiation is evaluated in this study (Fig. 2). It indicates the surgical incision into the tissue under the above mentioned conditions.

## Results

### Experiments in ambient air

**Fig. 3:**
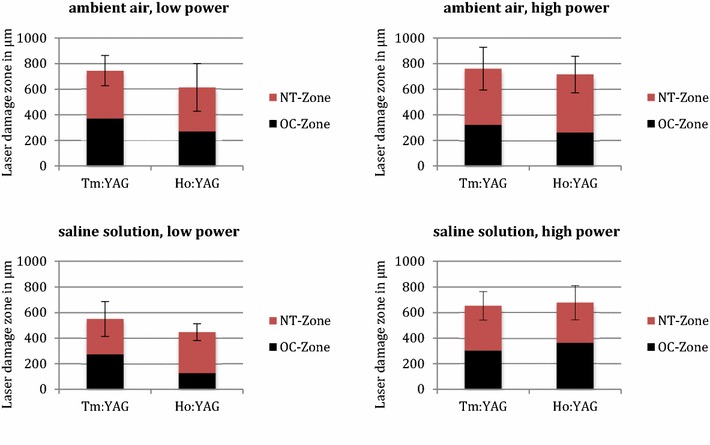
Laser damage zones of Tm:YAG and Ho:YAG

The low power setting (5 W) showed comparable laser damage zones for both lasers, amounting to 745 ± 119 µm for Tm:YAG and 614 ± 187 µm for Ho:YAG (Tables 1, 2). The high power setting experiments lead to laser damage zones of 760 ± 167 µm (Tm:YAG) versus 715 ± 142 µm (Ho:YAG) (Tables 3, 4). Thus the increase of laser power does not affect or increase the laser damage zone significantly in the ambient air (Fig. 3).

**Table 1:** Laser damage zone of Tm:YAG at 5 W in ambient air

	OC-zone (μm)	NT-zone (μm)	E-zone (μm)	Total (μm)	OC + NT (μm)	Inc. depth (μm)
A/K/R120/5W-1	382	348	298	1028	730	116
A/K/R120/5W-2	294	425	292	1011	719	465
A/K/R120/5W-4	440	346	257	1043	786	456
Average	372	373	282	1027	745	346
SD	74	45	22	16	119^a^	199

^a^The measurement error of OC + NT is the total of the standard deviations of the OC-zone plus NT-zone

**Table 2:** Laser damage zone of Ho:YAG at 0.5 J, 10 Hz, 5 W in ambient air

	OC-zone (μm)	NT-zone (μm)	E-zone (μm)	Total (μm)	OC + NT (μm)	Inc. depth (μm)
A/K/VPS/0.5,10-1	328	408	284	1020	736	0
A/K/VPS/0.5,10-3	308	400	450	1158	708	116
A/K/VPS/0.5,10-5	177	222	501	900	399	237
Average	271	343	412	1026	614	118
SD	82	105	113	129	189^a^	119

^a^The measurement error of OC + NT is the total of the standard deviations of the OC-zone plus NT-zone

**Table 3:** Laser damage zone of Tm:YAG at 120 W in ambient air

	OC-zone (μm)	NT-zone (μm)	E-zone (μm)	Total (μm)	OC + NT (μm)	Inc. depth (μm)
A/K/R120/120W-2	343	450	302	1095	793	5250
A/K/R120/120W-3	373	329	472	1174	702	5000
A/K/R120/120W-4	251	535	633	1419	786	5000
Average	322	438	469	1229	760	5083
SD	64	104	166	169	167^a^	144

^a^The measurement error of OC + NT is the total of the standard deviations of the OC-zone plus NT-zone

**Table 4:** Laser damage zone of Ho:YAG at 2 J, 40 Hz, 80 W in ambient air

	OC-zone (μm)	NT-zone (μm)	E-zone (μm)	Total (μm)	OC + NT (μm)	Inc. depth (μm)
A/KA/PS/2,40-2	215	434	434	1083	649	1432
A/K/VPS/2,40-3	247	378	654	1279	625	697
A/KA/PS/2,40-6	327	544	325	1196	871	1250
Average	263	452	471	1186	715	1126
SD	58	84	168	98	142^a^	383

^a^The measurement error of OC + NT is the total of the standard deviations of the OC-zone plus NT-zone

**Fig. 4:**
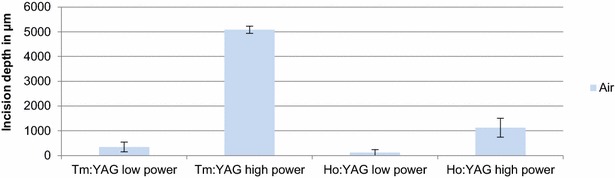
Incision depth of Tm:YAG and Ho:YAG for cutting in ambient air

The incision depth in ambient air on the low power setting was 346 ± 199 µm (Tm:YAG) and 118 ± 119 µm (Ho:YAG). On the high power setting, the incision depth was 5083 ± 144 versus 1126 ± 383 µm (Fig. 4). Therefore, the Tm:YAG laser is up to 4 times more efficient for incisions under the above mentioned conditions.

### Experiments in aqueous saline solution

In the saline solution experiments the laser damage zone on the low power setting amounts to 550 ± 137 µm for the Tm:YAG and 447 ± 65 µm for Ho:YAG (Tables 5, 6). On the high power setting we detected a laser damage zone of 653 ± 111 µm (Tm:YAG) and 677 ± 134 µm (Ho:YAG) (Tables 7, 8).

**Table 5:** Laser damage zone of Tm:YAG at 5 W in aqueous saline solution

	OC-zone (μm)	NT-zone (μm)	E-zone (μm)	Total (μm)	OC + NT (μm)	Inc. depth (μm)
Aq/K/R120/5W-3	255	221	344	820	476	0
Aq/K/R120/5W-5	355	260	343	958	615	0
Aq/K/R120/5W-6	212	346	309	867	558	0
Average	274	276	332	882	550	0
SD	73	64	20	70	137^a^	0

^a^The measurement error of OC + NT is the total of the standard deviations of the OC-zone plus NT-zone

**Table 6:** Laser damage zone of Ho:YAG at 0.5 J, 10 Hz, 5 W in aqueous saline solution

	OC-zone (μm)	NT-zone (μm)	E-zone (μm)	Total (μm)	OC + NT (μm)	Inc. depth (μm)
Aq/K/VPS/0.5.10-2	130	323	373	826	453	819
Aq/K/VPS/0.5,10-4	131	378	396	905	509	2231
Aq/K/VPS/0.5,10-6	116	264	420	800	380	593
Average	126	322	396	844	447	1214
SD	8	57	24	55	65^a^	888

^a^The measurement error of OC + NT is the total of the standard deviations of the OC-zone plus NT-zone

**Table 7:** Laser damage zone of Tm:YAG at 120 W in aqueous saline solution

	OC-zone (μm)	NT-zone (μm)	E-zone (μm)	Total (μm)	OC + NT (μm)	Inc. depth (μm)
Aq/K/R120/120W-1	318	330	350	998	648	5250
Aq/K/R12CV120W-5	274	275	393	942	549	3650
Aq/K/R12CV120W-6	320	442	373	1135	762	3250
Average	304	349	372	1025	653	4050
SD	26	85	22	99	111^a^	1058

^a^The measurement error of OC + NT is the total of the standard deviations of the OC-zone plus NT-zone

**Table 8:** Laser damage zone of Ho:YAG at 2 J, 40 Hz, 80 W in aqueous saline solution

	OC-zone (μm)	NT-zone (μm)	E-zone (μm)	Total (μm)	OC + NT (μm)	Inc. depth (μm)
Aq/K/VPS/2,40-1	278	292	432	1002	570	3500
Aq/K/VPS/2,40-4	321	318	600	1239	639	4500
Aq/K/VPS/2,40-5	497	326	719	1542	823	4250
Average	365	312	584	1261	677	4083
SD	116	18	144	271	134^a^	520

^a^The measurement error of OC + NT is the total of the standard deviations of the OC-zone plus NT-zone

Incision depth in saline solution on the low power setting amounts to Ho:YAG 1214 ± 888 µm. The Tm:YAG laser does not cut tissue at 5 W in saline solution.

**Fig. 5:**
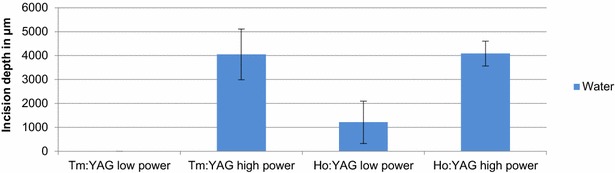
Incision depth of Tm:YAG and Ho:YAG for cutting in aqueous saline solution

The incision depth on the high power setting was 4050 ± 1058 µm (Tm:YAG) and 4083 ± 520 µm (Ho:YAG) (Fig. 5). Both lasers offer a comparable incision depth at their respective maximum power settings.

## Discussion

Several lasers were introduced in urological surgery and stone treatment during the recent past. One of the most widely researched laser devices is the Ho:YAG laser. Holmium laser enucleation of prostate (HoLEP) has become a comparable treatment option to TUR in BPH therapy with advantages in the treatment of large prostatic adenomas (Herrmann et al. 2012; Gilling et al. 2000, 2012). In addition, the Tm:YAG laser was introduced and showed convincing results for treatment of BPH in several studies (Bach et al. 2010; Netsch et al. 2012). Based on the different physical properties of each laser system, additional ex vivo experiments are necessary to understand the effect on tissue and to assess the complications and the clinical outcome after surgery.

The laser damage zone is defined by irreversible tissue damage due to laser irradiation (Bach et al. 2010). This damage zone is related but not identical to the absorption length at the laser wavelength in water. At the Tm:YAG laser wavelength of 2013 nm, the absorption length in water is 165 µm and approximately 426 µm at the Ho:YAG wavelength of 2123 nm (Irvine and Pollack 1968). Due to heat diffusion, the laser damage zone extends deeper into the irradiated tissue than the absorption length (Herrmann et al. 2012). The experiments described above reveal a laser damage zone into porcine kidney by the Tm:YAG laser of 550–760 μm—depending on the ambient medium (aqueous saline solution or ambient air). The laser power delivered to tissue has less influence on the extent of the laser damage zone than the ambient medium (aqueous saline solution or ambient air). The laser damage zone by the Ho:YAG laser (450–714 μm) compares favorably to the Tm:YAG laser. The present data is akin to the recently published examinations for porcine kidney (Bach et al. 2010; Kang et al. 2010).

The emission of the Ho:YAG laser is pulsed. Pulses of up to 6 kW peak power and approximately 250 μs duration are separated by more than 20–100 ms depending on the selected laser pulse repetition rate. The high laser pulse peak power of several kilowatts (kW) leads to explosive vaporization of tissue. Between laser pulses the generated heat dissipates into the surrounding tissue. The laser damage zone by the Ho:YAG laser in ambient air is slightly less than the laser damage zone by the continuously emitting Tm:YAG laser. The laser damage zone does not vary with the laser power, rather it relies on methodical variations such as translation speed or angulation of the laser fiber with respect to the tissue. The incision created by the Tm:YAG is smoother and deeper when compared to the Ho:YAG laser (Teichmann et al. 2007). The smoother Tm:YAG incision allows for simplified identification of anatomic landmarks than with incisions by the Ho:YAG laser. The smoother incisions by the Tm:YAG laser possibly translates into an easier learning curve for incising and resecting laser based techniques. This correlates with the rising interest in Tm:YAG based techniques for the treatment of the bladder outlet obstruction (BOO) due to benign prostatic enlargement (BPE) (Herrmann et al. 2010, 2012; Cui et al. 2014; Netsch et al. 2013; Wei et al. 2013).

A limitation of the study may be that experiments were not carried out in a perfused kidney model as in some other studies. The present experiments were performed on ex vivo porcine kidneys as a model for prostate surgery. Recent studies confirmed that a perfused ex vivo kidney model is a consistent model for prostate surgery (Michel et al. 1996). Studies comparing different therapy modalities such as bipolar electro cautery, diode-, KTP- and thulium-lasers to evaluate the laser damage zones and hemostatic effects of these surgery devices were performed (Heinrich et al. 2010; Wendt-Nordahl et al. 2008; Michel et al. 1996; Wendt-Nordahl et al. 2007). In the present experiments we used a non-perfused kidney model as a model for prostate surgery. It was shown that none-perfused kidneys are sufficient for the examination of laser damage zones as safety criteria for laser surgery because the laser damage zones in perfused and none perfused porcine kidneys are similar (Khoder et al. 2012). The in vivo effects may be different, especially for laser use on prostate tissue.

Another limitation of the present study is that any comparison of a pulsed laser with a continuous wave laser is very difficult due to fundamental differences in laser beam tissue interaction by the properties of heat conduction and heat excess—thermal relaxation time.

A further limitation of the study is that all variances in tissue composition with regard to waterlevel can alter the immediate tissue effect and lead to different results for laser effect. We tried to consider this fact by comparing the arithmetic mean of three equal experiments for each setting.

## Conclusion

Our study demonstrates that Tm:YAG lasers and Ho:YAG lasers emitting laser radiation at approximately 2 µm share similar properties with regard to tissue interaction, whereas Tm:YAG being a continuous wave laser could demonstrate higher capacity for vaporization when compared to Ho:YAG. This translates into better performance in resecting and vaporizing approaches for soft tissue surgery. Both create similar necrotic and outer coagulation zones. The laser damage zone is about 1 mm and similar for both laser generators. These in vitro studies correspond well with histologic measurements for in vivo measurements in prostate and kidney.

The Ho:YAG has proved to be a versatile laser in urology. The Tm:YAG laser offers equivalent tissue cutting performance, hemostasis, and safety for soft tissue applications while also providing higher vaporization capacities. The Tm:YAG is a safe, effective and reliable surgical device for many applications in urology and other surgical disciplines.

## Abbreviations

Tm:YAG: thulium yttrium aluminium garnet
Ho:YAG: holmium yttrium aluminium garnet
W: watt
kW: kilowatt
J: joule
Hz: hertz
mm: millimeter
cm: centimeter
µm: micrometer
nm: nanometer
KTP: potassium-titanyl-phosphate-laser
LBO: lithium-borate-laser
°C: degree celsius
s: second
ms: millisecond
µs: microsecond
h: hour
NaCl: sodium chloride
E-zone: edema zone
OC-zone: outer coagulation zone
NT-zone: necrotic tissue zone
HoLEP: holmium laser enucleation of the prostate
TUR: trans-urethral resection
BPH: benign prostatic hyperplasia
BOO: bladder outlet obstruction
BPE: benign prostate enlargement

## References

[CR1] Bach T, Herrmann TR, Ganzer R, Burchardt M, Gross AJ (2007). RevoLix vaporesection of the prostate: initial results of 54 patients with a 1-year follow-up. World J Urol.

[CR2] Bach T (2010). 70 versus 120 W thulium:yttrium-aluminium-garnet 2 micron continuous-wave laser for the treatment of benign prostatic hyperplasia: a systematic ex vivo evaluation. BJU Int.

[CR3] Cui D (2014). A randomized trial comparing thulium laser resection to standard transurethral resection of the prostate for symptomatic benign prostatic hyperplasia: four-year follow-up results. World J Urol.

[CR4] Fried NM, Murray KE (2005). High-power thulium fiber laser ablation of urinary tissues at 1.94 microm. J Endourol.

[CR5] Gilling PJ, Kennett KM, Fraundorfer MR (2000). Holmium laser enucleation of the prostate for glands larger than 100 g: an endourologic alternative to open prostatectomy. J Endourol.

[CR6] Gilling PJ (2012). Long-term results of a randomized trial comparing holmium laser enucleation of the prostate and transurethral resection of the prostate: results at 7 years. BJU Int.

[CR7] Gupta PK (2007). Is the holmium:YAG laser the best intracorporeal lithotripter for the ureter? A 3-year retrospective study. J Endourol.

[CR8] Heinrich E (2010). 120 W lithium triborate laser for photoselective vaporization of the prostate: comparison with 80 W potassium-titanyl-phosphate laser in an ex vivo model. J Endourol.

[CR9] Herrmann TR (2010). Thulium laser enucleation of the prostate (ThuLEP): transurethral anatomical prostatectomy with laser support. Introduction of a novel technique for the treatment of benign prostatic obstruction. World J Urol.

[CR10] Herrmann TR, Liatsikos EN, Nagele U, Traxer O, Merseburger AS (2012). EAU guidelines on laser technologies. Eur Urol.

[CR11] Irvine WM, Pollack JB (1968). Infrared optical properties of water and ice spheres. Icarus.

[CR12] Kang HW, Kim J, Peng YS (2010). In vitro investigation of wavelength-dependent tissue ablation: laser prostatectomy between 532 nm and 2.01 micron. Lasers Surg Med.

[CR13] Khoder WY (2012). Ex vivo comparison of the tissue effects of six laser wavelengths for potential use in laser supported partial nephrectomy. J Biomed Opt.

[CR14] Michel MS, Kohrmann KU, Weber A, Krautschick AW, Alken P (1996). Rotoresect: new technique for resection of the prostate: experimental phase. J Endourol.

[CR15] Netsch C, Bach T, Herrmann TR, Gross AJ (2012). Thulium:YAG VapoEnucleation of the prostate in large glands: a prospective comparison using 70- and 120-W 2-micron lasers. Asian J Androl.

[CR16] Netsch C, Bach T, Herrmann TR, Neubauer O, Gross AJ (2013). Evaluation of the learning curve for Thulium VapoEnucleation of the prostate (ThuVEP) using a mentor-based approach. World J Urol.

[CR17] Pierre S, Preminger GM (2007). Holmium laser for stone management. World J Urol.

[CR18] Teichmann HO, Herrmann TR, Bach T (2007). Technical aspects of lasers in urology. World J Urol.

[CR19] Wendt-Nordahl G (2007). 980-nm diode laser: a novel laser technology for vaporization of the prostate. Eur Urol.

[CR20] Wendt-Nordahl G (2008). Systematic evaluation of a recently introduced 2-micron continuous-wave thulium laser for vaporesection of the prostate. J Endourol.

[CR21] Wei H et al (2013) Thulium laser resection versus plasmakinetic resection of prostates larger than 80 ml. World J Urol 32(4):1077–1085. doi:10.1007/s00345-013-1210-4 10.1007/s00345-013-1210-4 24264126

